# Perioperative Hyperoxyphobia: Justified or Not? Benefits and Harms of Hyperoxia during Surgery

**DOI:** 10.3390/jcm9030642

**Published:** 2020-02-28

**Authors:** Robert P. Weenink, Stijn W. de Jonge, Robert A. van Hulst, Thijs T. Wingelaar, Pieter-Jan A. M. van Ooij, Rogier V. Immink, Benedikt Preckel, Markus W. Hollmann

**Affiliations:** 1 Department of Anaesthesia, Amsterdam UMC, University of Amsterdam, 1105 AZ, Amsterdam, The Netherlands; 2 Department of Surgery, Amsterdam UMC, University of Amsterdam, 1105 AZ, Amsterdam, The Netherlands; 3 Department of Hyperbaric medicine, Amsterdam UMC, University of Amsterdam, 1105 AZ, Amsterdam, The Netherlands; 4 Diving Medical Center, Royal Netherlands Navy, 1780 CA, Den Helder, The Netherlands; 5 Department of Pulmonology, Amsterdam UMC, University of Amsterdam, 1105 AZ, Amsterdam, The Netherlands

**Keywords:** hyperbaric oxygenation, hyperoxia, oxygen toxicity, perioperative care, reactive oxygen species, surgical wound infection

## Abstract

The use of an inspiratory oxygen fraction of 0.80 during surgery is a topic of ongoing debate. Opponents claim that increased oxidative stress, atelectasis, and impaired oxygen delivery due to hyperoxic vasoconstriction are detrimental. Proponents point to the beneficial effects on the incidence of surgical site infections and postoperative nausea and vomiting. Also, hyperoxygenation is thought to extend the safety margin in case of acute intraoperative emergencies. This review provides a comprehensive risk-benefit analysis for the use of perioperative hyperoxia in noncritically ill adults based on clinical evidence and supported by physiological deduction where needed. Data from the field of hyperbaric medicine, as a model of extreme hyperoxygenation, are extrapolated to the perioperative setting. We ultimately conclude that current evidence is in favour of hyperoxia in noncritically ill intubated adult surgical patients.

## 1. Introduction

Perioperative use of an inspiratory oxygen fraction (FiO_2_) of 0.80 (hyperoxia) is controversial, and at times, is even emotionally discussed in the fields of anaesthesia and surgery. The 2016 World Health Organisation guidelines on prevention of surgical site infections (SSI) recommended the use of a FiO_2_ of 0.80 in intubated patients undergoing surgery [1]. This guideline, developed without the involvement of anaesthetists, added to the discussion and elicited several letters from around the world criticising its conclusions [2,3,4,5,6], especially when some of the trials demonstrating a positive effect on SSI were retracted or came under scrutiny [7,8,9,10]. Recently, the World Health Organisation updated its guidelines, (now also with the support of anaesthetists), based on a new systematic review excluding the questionable trials [11], and an independent review specifically focussing on potential adverse effects of hyperoxia [12]. Although the strength of the recommendation was changed from strong to conditional, the general advice to ventilate intubated surgical patients with 0.80 FiO_2_ was upheld [13].

In the following article, we give a data-supported risk-benefit analysis for the use of 0.80 FiO_2_ in noncritically ill patients in the perioperative setting. Firstly, we will examine the supposed adverse effects of perioperative hyperoxia, namely increased mortality, increased oxidative stress due to reactive oxygen species (ROS), hyperoxic vasoconstriction leading to impairment of tissue oxygenation, and atelectasis. We will start by exploring data available from clinical trials, continuing with a physiological deduction to elucidate in more detail whether a potential for harm could exist, and lastly, we address the ultimate form of hyperoxia in clinical practice, namely hyperbaric oxygen therapy (HBOT). After discussing the possible adverse effects from these three perspectives, we will explore the suggested positive effects of perioperative hyperoxia: decreased incidence of SSI, decreased incidence of postoperative nausea and vomiting (PONV), and increased margin of safety in acute intraoperative emergencies.

## 2. Adverse Effects

### 2.1. Data from Clinical Studies

The suggested adverse effects of hyperoxia are increased oxidative stress due to ROS, hyperoxic vasoconstriction, and resorption atelectasis. Clinically, these may lead to increased mortality, pulmonary complications, and cardio- and cerebrovascular complications. We will, therefore, analyse data on adverse event (AE) rates from clinical trials on perioperative hyperoxia, bearing in mind that none of these trials were designed to detect differences in the incidence of AE.

#### 2.1.1. Mortality

We will first examine mortality, as the ultimate clinical endpoint of any adverse effect. Interestingly, the point estimates of all but two [14,15] of the clinical trials on perioperative high versus normal FiO_2_ indicated lower, rather than higher, short-term mortality in patients given 0.80 FiO_2_ [16,17,18,19,20]. However, each of these trials had low mortality rates with wide confidence intervals, including unity. A recent systematic review and meta-analysis indicated that there was no association between high perioperative FiO_2_ and short-term mortality (relative risk; RR = 0.49, 95% CI; 0.17–1.37) or long-term mortality (RR = 0.96, 95% CI; 0.65–1.42) [12].

In contrast to the evidence from randomised trials, a retrospective observational study showed an increased risk of mortality within 30 days after administration of high versus normal FiO_2_ (odds ratio = 1.97, 95% CI; 1.30–2.99) [21]. A nonrandomised prospective study found a comparable relative risk but a much wider confidence interval (RR = 1.97, 95% CI; 0.71–5.47) [22]. However, outside of randomised clinical trials, the use of high FiO_2_ is not random but generally used in sicker patients, and interpretation of these data is therefore challenging. The first study [21] was limited by indications of residual confounding, exclusion of more than 35,000 potentially eligible participants, and deviation from the predefined outcome in the final report. The second study [22] was based on registry and billing data, lacked blinding and also deviated from the predefined outcome in the final report.

The lack of difference in mortality in trials on perioperative hyperoxia is in contrast to studies on critically ill patients. Chu and colleagues recently reported higher mortality in critically ill adults after exposure to liberal oxygen therapy in a meta-analysis of randomised controlled trials [23]. These findings led to revised guidelines for oxygen therapy for acutely ill medical patients [24] and, indirectly, also raised concerns on the safety of perioperative hyperoxia. However, the data reported by Chu and colleagues, although important for the populations studied, are not suited for extrapolation to perioperative hyperoxia. The population described—consisting of patients in the acute phase after stroke, myocardial infarction, and other life-threatening events that require acute critical care—is not comparable to patients deemed fit for elective surgery. Moreover, Chu and colleagues studied a different intervention. They defined liberal oxygen as any oxygen target higher than that of the control group; interventions typically remained between 0.30 and 0.40 FiO_2_ [23]. In general anaesthesia this is considered the range required to overcome the pathophysiological respiratory changes of anaesthesia and maintain adequate oxygen saturation [25]. Therefore, in research on perioperative hyperoxia, it is considered the conservative control and contrasted to 0.80 FiO_2_ [11]. In addition, in the studies included by Chu and colleagues, exposure to hyperoxia was typically more than 12 h and ranged up to a full week, while perioperative hyperoxia rarely exceeds a few hours. All in all, hyperoxia studies outside of the operating room were done in a different population and using a different intervention, and it does not, therefore, seem reasonable to extrapolate these findings to the surgical patient.

#### 2.1.2. Postoperative Pulmonary Complications

The most likely clinical consequence of pulmonary oxygen toxicity due to ROS, as well as resorption atelectasis, would be an increased incidence of postoperative pulmonary complications. Multiple studies report on adverse effects such as pneumonia or composite measures of respiratory complications [14,15,18,26]. A meta-analysis of all but the most recent of these studies demonstrated no increase in respiratory AE after 30 min to 4 h perioperative administration of 0.80 versus 0.30–0.35 FiO_2_, nor was there a difference in the incidence of postoperative atelectasis [12]. A post-hoc analysis [27] of over 5000 patients enrolled in a cohort study [22] confirmed that perioperative 0.80 FiO_2_ does not worsen pulmonary outcome.

#### 2.1.3. Cardiovascular and Cerebrovascular Complications

It has been theorised that hyperoxic vasoconstriction may lead to decreased tissue oxygenation [28,29,30], which might culminate in increased rates of myocardial infarction or stroke in patients treated with high perioperative FiO_2_. However, when regarding cardiovascular AE, a meta-analysis of three studies showed no signal of increased ratios of these AE in patients receiving high FiO_2_ [12]. Data on the incidence of stroke and transitory cerebral ischemia are available from only one trial; no increased incidence was found in patients who received high instead of normal FiO_2_ [31].

### 2.2. Pathophysiological Effects of Hyperoxia

Clinical data did not show a signal of harm due to perioperative hyperoxia. We will now examine whether harmful effects of hyperoxia are likely to exist, based on pathophysiological deduction.

#### 2.2.1. Oxidative Stress

Oxygen is a stable molecule that by itself, is not considered toxic [32]. However, it can easily gain an electron, creating the superoxide radical, a ROS that acts as a precursor to most of the other ROS. ROS are highly reactive molecules, capable of damaging proteins, lipids, and DNA, which may ultimately lead to apoptosis [33]. Even when breathing 0.21 FiO_2_, there is a continuous production of ROS, against which cells have extensive antioxidative defence mechanisms [34]. Oxidative stress can be regarded as a misbalance between ROS production and antioxidative mechanisms. Despite the fact that oxidative stress is thought to be the common denominator in various pathological processes [35], studies in healthy humans that aimed to quantify oxidative stress and (anti)inflammatory effects of hyperoxia have yielded conflicting results. In a study in which healthy volunteers breathed 1.0 FiO_2_ for 3.5 h, no increase in neutrophil intracellular ROS generation and cytokine production was observed [36]. In contrast, systemic oxidative stress, quantified using plasma malondialdehyde concentration, was demonstrated to increase after exposure to 30 min [37] or 2 h [38] of 1.0 FiO_2_ in two studies, and after the administration of 0.80 FiO_2_ during abdominal surgery [39]. One of these studies also showed a decrease in plasma nitrite concentration after exposure to 30 min of 1.0 FiO_2_, demonstrating reduced bioavailability of nitric oxide caused by increased oxidative stress [37].

When specifically assessing the toxic effects of hyperoxia on lung tissue, some studies have demonstrated signs of pulmonary lipid peroxidation and inflammation due to normobaric hyperoxia. For instance, elevations of volatile exhaled markers of lipid peroxidation such as ethane, pentane, and methylated alkanes have been found after breathing 1.0 FiO_2_ for 2 h [38,40,41]. Studies on alveolar inflammation, quantified using the measurement of exhaled nitric oxide concentration (FeNO), showed conflicting results. In one study an increase in FeNO was found after breathing 1.0 FiO_2_ for 10 min [41], while two other studies measured a decrease in FeNO when breathing 1.0 FiO_2_ for 90 min [42,43]. These differences in FeNO values are most likely due to technical variations in collection methods of FeNO between the studies [44].

#### 2.2.2. Vasoconstriction and Cellular Oxygen Delivery

Some authors have hypothesised that arterial hyperoxaemia may lead to paradoxical tissue hypoxia through hyperoxic vasoconstriction [28,29,30]. Hyperoxia indeed causes arteriolar vasoconstriction in many vascular beds, including the coronary and cerebral circulation [45]; this was suggested to be a normal metabolic autoregulatory phenomenon, protection against oxidative stress by ROS, or a combination of both [45]. Hyperoxic vasoconstriction increases peripheral vascular resistance [46,47], increases parasympathetic outflow [48], and modestly decreases sympathetic outflow [49,50]. The effects of hyperoxia in healthy volunteers have been summarised by Smit and colleagues: systemic vascular resistance increases 12% (95% CI; 8.6–16), heart rate decreases 6.5% (95% CI; 5.0–8.1), mean arterial pressure increases 2.0% (95% CI; 0.2–3.9), stroke volume decreases 3.0% (95%CI [0.3–5.7]) and cardiac output decreases 10% (95% CI; 7.3–13) [28]. Other studies found that cerebral blood flow decreased by 13% [51], and coronary vascular resistance increased by 30% [52]. Of note, hyperoxia is not a universal vasoconstrictor; for instance, in the pulmonary vasculature it results in vasodilation.

When FiO_2_ increases fivefold from 0.21 to 1.0, arterial oxygen content increases only approximately 7%; this is because most oxygen is bound to haemoglobin, which is already almost maximally saturated at 0.21 FiO_2_. Since hyperoxia decreases perfusion and only slightly increases oxygen content, some authors have hypothesised that hyperoxia impairs tissue oxygenation [28,29,30]. This hypothesis, however, is not supported by animal and human experiments. In pigs subjected to increasing FiO_2_, both cerebral and myocardial tissue oxygen tension (PtO_2_) increased proportionally with increasing FiO_2_ [53,54]. When pigs were subjected to haemodilution until their electrocardiogram showed ischaemic changes, hyperoxia, although it caused coronary vasoconstriction and reduced coronary blood flow, preserved myocardial oxygenation and improved electrocardiogram abnormalities [55]. A randomised controlled trial in colorectal surgery demonstrated a twofold increase in muscular and subcutaneous PtO_2_ in patients breathing 0.80 versus 0.30 FiO_2_ [17].

In conclusion, available evidence suggests that hyperoxia does not impair tissue oxygen delivery [56], but contrarily (despite vasoconstrictive properties), increases tissue oxygenation. Additionally, hyperoxia improves the efficiency of the mitochondrial respiratory chain and increases the respiratory quotient to values near 1.0 (indicating a shift to pure carbohydrate metabolism) [57,58,59]. This decreases oxygen demand and is responsible for the observed decrease in oxygen uptake during hyperoxia [60,61].

The aforementioned effects of hyperoxia have mostly been studied in awake patients, and extrapolation to the anaesthetised patient should be done with caution. The interaction of hemodynamic compromise induced by anaesthesia and hyperoxic vasoconstriction is hard to predict. It has been suggested that hyperoxic vasoconstriction may reduce the need for pharmacological vasopression during anaesthesia [59]. On the other hand, the combination of anaesthetic induced haemodynamic compromise and hyperoxic vasoconstriction could theoretically increase the risk of stroke or other perfusion-related complications. Additionally, when it comes to cerebral perfusion, the effect of arterial carbon dioxide tension (PaCO_2_), or more precisely, the change in blood pH, has to be taken into account. Awake subjects increase their minute ventilation by approximately 10% when subjected to hyperoxia, resulting in a 0.4 kPa decrease in PaCO_2_ [62]. The ensuing hypocarbic vasoconstriction will add to the vasoconstriction caused by hyperoxia. In patients under general anaesthesia, minute ventilation and thereby PaCO_2_ are controlled by the anaesthetist. Cerebral vessels are much more responsive to pH changes elicited by PaCO_2_ changes, than to changes in arterial oxygen tension (PaO_2_) [63], and it is, therefore, reasonable to assume that the impact of minute ventilation as determined by the anaesthetist is of much greater importance to cerebral blood flow than any changes caused by hyperoxia.

#### 2.2.3. Atelectasis

Data from clinical trials demonstrated no increase in the incidence of postoperative atelectasis or other pulmonary complications after the use of perioperative high FiO_2_, but AE, as reported in trials, may be too crude a method to reliably determine the incidence of atelectasis and its possible negative consequences. We will, therefore, briefly explore the physiology behind resorption atelectasis and its prevention.

Hyperoxia can cause absorption atelectasis [64], which may increase pulmonary shunting [65]. In healthy subjects breathing 1.0 FiO_2_, atelectasis combined with inhibition of hypoxic pulmonary vasoconstriction doubles intrapulmonary right-to-left shunt, compared to subjects breathing room air [59]. However, lowering FiO_2_ to 0.80 reduces atelectasis substantially: in a study where 0.80 FiO_2_ was used during anaesthesia induction, the atelectatic area in the basal lung fields as measured by computed tomography was reduced from 9.8 ± 5.2 cm^2^ (1.0 FiO_2_) to 1.3 ± 1.2 cm^2^ (0.8 FiO_2_) [64]. Moreover, application of a positive end-expiratory pressure of 10 cm H_2_O in intubated patients effectively prevents atelectasis [66]. Therefore, during surgery atelectasis due to 0.80 FiO_2_ combined with positive end-expiratory pressure is not a great concern.

Irrespective of the FiO_2_ used during the course of surgery, most anaesthetists routinely apply high (0.80–1.0) FiO_2_ towards the end of anaesthesia, to provide a safety margin in case of respiratory problems after extubation. In contrary to the intubated patient, however, positive pressure ventilation is less easily applied in extubated patients, and resorption atelectasis is, therefore, most likely to be clinically relevant in the extubated patient shortly after surgery. Again, applying 0.80 instead of 1.0 FiO_2_ will greatly diminish atelectatic volume. A balance between providing an optimal margin of safety during extubation, and prevention of atelectasis, might, therefore, be reached by applying 0.80 FiO_2_.

### 2.3. Hyperbaric Oxygenation

We now look at the most severe form of hyperoxia, namely HBOT. We believe that a lack of harm of hyperbaric hyperoxia would make adverse effects of a short-term perioperative normobaric hyperoxia even less likely.

Patients undergoing HBOT breathe 1.0 FiO_2_ at pressures of 1.4–2.8 atmospheres absolute (ATA) (142–284 kPa) for varying amounts of time [67]. Clinically employed regimens may involve 20–80 daily sessions at 2.5 ATA (253 kPa) of 90–120 min each, for instance for the treatment of diabetic ulcers and late radiation tissue injuries [68]. Given these indications for HBOT, the medical condition of these patients regarding comorbidity can be considered quite similar to that of the general surgical population. PaO_2_ in HBOT is up to 3.5 times higher than during normobaric 0.80 FiO_2_, and PtO_2_ during HBOT may be as high as 55 kPa [69], as opposed to values in the range of 4.0–6.7 kPa with normobaric 0.21 FiO_2_ [70]. Despite these high PtO_2_ values, large retrospective series show that HBOT is safe [71]. In hyperbaric medicine, the increased production of ROS is considered to have positive aspects. ROS serve as signalling molecules for cytokines and hormones, and stimulate stem cells and growth factors, resulting in neovascularisation and wound healing in chronic diabetic wounds [72,73]. Additionally, HBOT inhibits neutrophil adherence in reperfusion injury and therefore has the potential to improve postischemic tissue survival [74]. Regarding pulmonary toxicity, recent experiments from our group in healthy subjects exposed to six daily HBOT sessions of 90 min at 2.5 ATA (253 kPa) showed no significant changes in any of the measured pulmonary function tests, including diffusion capacity and alveolar volume [75], suggesting that even in HBOT, pulmonary oxygen toxicity rarely reaches a level of clinical significance. Cerebral oxygen toxicity is a known rare complication of HBOT, occurring at partial oxygen tensions above 1.6 ATA (162 kPa) [76] at a rate of 1:40.000–1:60.000 exposures [77,78]. Lastly, the fact that HBOT provides vasoconstriction while increasing PtO_2_ is used as a beneficial effect, for instance, to reduce intracranial pressure in traumatic brain injury [79] and cases of cerebral arterial gas embolism [80].

## 3. Beneficial Effects

### 3.1. Decreased Incidence of Surgical Site Infections

The most widely used argument for the use of high FiO_2_ perioperatively is the reduction of the risk of SSI. Surgical wounds are poorly oxygenated, and low oxygen tension is associated with SSI [81,82]. Hence, increasing PtO_2_ at the surgical wound by providing increased FiO_2_ might exert beneficial effects [82]. Although oxygen delivery is much more efficiently increased by enhancing cardiac output, this does not necessarily increase PtO_2_. The most efficient way of increasing PtO_2_ to supranormal levels and thereby recruiting the antibacterial effect of oxygen in the tissue is by increasing PaO_2_ through the increase of FiO_2_ [17], which occurs despite hyperoxic vasoconstriction and reduction of cardiac output, as discussed above.

The first large trial to test the hypothesis that hyperoxia may reduce SSI showed promising results; patients randomised to receive a 0.80 FiO_2_ perioperatively had a 50% reduction in SSI compared to patients who received the standard 0.30 FiO_2_ [17]. This trial was well designed, with double-blind outcome assessment limited to culture proven infections, and assessment of oxygen delivery by measurement of PaO_2_ and PtO_2_. Subsequent trials did not meet this standard and could not consistently reproduce these results [14,15,16,20]. The issue was further complicated when concerns on the validity of some of the trials arose [7,8,9,10]. A large nonrandomised study in which default operating room FiO_2_ settings varied biweekly between 0.30 and 0.80 did not show a beneficial effect of hyperoxia on a composite outcome of infectious complications [22]. However, interpretation of these data was challenged by the lack of randomisation and blinding, reliance on registry and billing data for outcome assessment, contamination between the two treatment groups, and deviation from the predefined primary outcome. Additionally, the study did not report total SSI, which makes it difficult to compare the results to other studies. A recent meta-analysis of randomised controlled trials that excluded the trials under scrutiny and nonrandomised studies found no definite benefit of the use of high FiO_2_ overall but did find evidence for a protective effect in patients under general anaesthesia with tracheal intubation [11]. Conversely, there was no effect of high FiO_2_ on SSI in patients who were awake and received increased FiO_2_ through a facemask.

Several explanations have been proposed for the differences in the effects of hyperoxia on SSI in these trials, including the inadequacy of oxygen delivery through a facemask, and differences in the use of general anaesthesia, mechanical ventilation, laparoscopic surgery, fluid regimen, temperature control, and pain management. For instance, the trials delivering oxygen through a facemask were exclusively performed on young female patients undergoing caesarean section under spinal anaesthesia. This short-lasting surgery in mostly young and healthy females having pregnancy-related changes of lung physiology should not be directly compared to the intubated, mostly older patients with relevant comorbidities undergoing longer-lasting abdominal surgery.

Many of the explanations for the observed difference in efficacy of hyperoxia on SSI rely on the idea that the application of high FiO_2_ alone does not guarantee adequate tissue oxygenation. Adequate gas exchange, as well as sufficient macro- and microcirculatory flow, are needed to actually deliver oxygen to the tissues; this is also suggested by the results of the most recent trial on perioperative hyperoxia, published after the aforementioned systematic review, that showed no beneficial effect of 0.80 versus 0.30 FiO_2_ in abdominal surgery [15]. In this study, both groups were ventilated using lung-protective settings, including modulation of end-expiratory pressure to optimise pulmonary compliance. Other confounding factors may include fluid regimen and the use of vasopressors. For instance, experimental work showed that epinephrine infusion in healthy volunteers makes PtO_2_ unresponsive to increases in PaO_2_ [83]. Modern, more restricted fluid regimens inevitably increase the use of vasopressors. Important negative trials were conducted using restricted fluid regimens [14,20], and increasingly included laparoscopic surgery [20], whereas important positive trials hydrated participants liberally and yielded exclusively at laparotomies [16,17]. Only the first trial [17] measured PtO_2_ and found a steep increase in subcutaneous and muscle PtO_2_, as well as PaO_2_ alongside the decrease in SSI. For all the other trials, it is not known if the intervention accomplished increased tissue oxygenation.

### 3.2. Decreased Incidence of Postoperative Nausea and Vomiting

PONV remains a troublesome complication of general anaesthesia. When properly and routinely applied, conventional pharmacological measures provide a large degree of protection against PONV [84]. Intraoperative use of high FiO_2_ has antiemetic properties. Hovaguimian and colleagues analysed eight trials on high versus normal perioperative FiO_2_ in patients subjected to inhalational (volatile) anaesthesia without antiemetic prophylaxis. Patients receiving high FiO_2_ had a RR of 0.75 (95% CI; 0.62–0.90) for nausea in the first 24 h postoperatively, and a RR of 0.72 (95% CI; 0.56–0.92) for vomiting in the first 24 h postoperatively, when compared to patients receiving normal FiO_2_ [85]. Although the effects were smaller and mostly nonsignificant when patients receiving propofol anaesthesia and/or antiemetic prophylaxis were included, the high availability and low cost of oxygen make hyperoxia a suitable addition to the antiemetic armamentarium of the anaesthetist.

### 3.3. Increased Safety Margin in Cases of Intraoperative Emergencies

Preoxygenation with 1.0 FiO_2_ is recommended for all patients during induction of anaesthesia [86], to increase the time until desaturation in case of a “cannot intubate, cannot oxygenate” scenario, which nowadays occurs in less than 0.01% of anaesthetic inductions [87]. In healthy volunteers, 1.0 FiO_2_ provides a margin of safety of up to about 8 min until oxygen desaturation of haemoglobin to 90% [88]. In almost all unexpected AE during surgery, one of the promptly taken measures is to increase FiO_2_ to 1.0, to buy time to deal with the emergency [89]; this is especially useful during massive bleeding since the relative contribution of physically dissolved oxygen to total oxygen content increases tremendously when haemoglobin has been lost and replaced by other fluids [90]. Unfortunately, in many relevant AE (e.g., sudden massive haemorrhage, accidental extubation), the anaesthetist does not have the time to respond and adequately fill the patient’s functional residual capacity with oxygen.

No data exist on the incidence of intraoperative AE requiring the application of high FiO_2_, but an estimate can be calculated based on a recent study that reports AE in two United States hospitals [91]. The overall rate of intraoperative AE after initiation of a mandatory reporting system was 0.64% in one hospital, and 1.36% in the other. When analysing the categories of reported AE, a safe estimate is that high FiO_2_ is likely to have been applied by the anaesthetist in 35%–60% of AE, as part of handling the AE, which would mean that of all of the surgical cases in these two hospitals throughout the study period, 0.23%–0.83% would have required the use of high FiO_2_ to respond to an intraoperative AE. This percentage is much higher than the incidence of “cannot intubate, cannot oxygenate” during induction. Therefore, since all our patients deserve preoxygenation to increase the margin of safety in case of the highly unlikely scenario of ‘cannot intubate, cannot oxygenate’, we believe our patients also deserve high FiO_2_ during the rest of the procedure, to prepare for much more likely intraoperative AE’s.

## 4. Conclusions

In this article, we have reviewed supposed harms and benefits of perioperative hyperoxia. We have demonstrated that although perioperative hyperoxia may lead to quantifiable oxidative stress, this does not translate into increased postoperative pulmonary complications or mortality. Furthermore, hyperoxic vasoconstriction does not lead to decreased tissue oxygenation, nor increased cardiovascular or cerebrovascular complications. Atelectasis is not a concern in the intubated patient treated with positive end-expiratory pressure. Data from the field of hyperbaric medicine indicate that even repeated administration of 1.0 FiO_2_ under hyperbaric conditions is safe. On the other hand, hyperoxia in intubated surgical patients reduces SSI. In addition, hyperoxia has an antiemetic effect and provides a margin of safety in case of intraoperative emergencies.

Given the lack of indication of harm of perioperative hyperoxygenation and the solid beneficial effects to be gained, we recommend providing 0.80 FiO_2_ to noncritically ill, intubated adults during surgery. Future studies should look into determinants of tissue oxygenation other than FiO_2_ and should include measurement of PtO_2_ to verify that interventions actually achieve the desired goal. Randomised trials should look carefully into a wide spectrum of possible AE’s.
